# Dissipation and Residues of Pyraclostrobin in *Rosa roxburghii* and Soil under Filed Conditions

**DOI:** 10.3390/foods11050669

**Published:** 2022-02-24

**Authors:** Lei Han, Qiong Wu, Xiaomao Wu

**Affiliations:** 1 Institute of Crop Protection, Guizhou University, Guiyang 550025, China; lhan925@sina.com; 2 Provincial Key Laboratory for Agricultural Pest Management in Mountainous Region, Guizhou University, Guiyang 550025, China; 3 Plant Protection and Plant Inspection Station of Guizhou Province, Guiyang 550001, China; wuqiong2008@163.com

**Keywords:** pyraclostrobin, *Rosa roxburghii*, soil, dissipation, residue

## Abstract

*Rosa roxburghii* has been widely planted in China. Powdery mildew is the most serious disease of *R. roxburghii* cultivation. Pyraclostrobin was widely used as a novel fungicide to control powdery mildew of *R. roxburghii*. To assess the safety of pyraclostrobin for use on *R. roxburghii* fruits, its residue rapid analysis as well as an investigation on its dissipation behaviors and terminal residues in *R. roxburghii* and soil under field conditions were carried out. The QuEChERS method was simplified using LC–MS/MS detection and combined with liquid–liquid extraction purification to allow determination of pyraclostrobin levels in *R. roxburghii* fruits and the soil. The fortified recoveries at 0.1~5.0 mg/kg were 93.48~102.48%, with the relative standard deviation of 0.64~3.21%. The limit of detection of the analytical method was 0.16 and 0.15 µg/kg for *R. roxburghii* fruit and soil, respectively. The effects of different spray equipment and formulations on the persistence of pyraclostrobin in *R.* *roxburghii* were as follows: gaston gasoline piggyback agricultural sprayer (5.38 d) > manual agricultural backpack sprayer (3.37 d) > knapsack multi-function electric sprayer (2.91 d), suspension concentrate (SC) (6.78 d) > wettable powder (WP) (5.64 d) > water dispersible granule (WG) (4.69 d). The degradation of pyraclostrobin followed the first-order kinetics and its half-lives in *R.* *roxburghii* and soil were 6.20~7.79 days and 3.86~5.95 days, respectively. The terminal residues of pyraclostrobin in *R*. *roxburghii* and soil were 0.169~1.236 mg/kg and 0.105~3.153 mg/kg, respectively. This study provides data for the establishment of the maximum residue limit (MRL) as well as the safe and rational use of pyraclostrobin in *R*. *roxburghii* production.

## 1. Introduction

*Rosa roxburghii*, as a popular new fruit, is rich in vitamin C, flavonoids, superoxide dismutase (SOD) and various minerals, which can improve human immunity and reduce the risk of cancer [1,2]. However, *R. roxburghii* quality and yield are often severely reduced due to various diseases such as powdery mildew, brown spot, sooty mold, stem rot and virus disease. Powdery mildew is one of most serious diseases of *R. roxburghii* in production. To reduce economic loss caused by diseases, various pesticides are used in *R*. *roxburghii* cultivation, of whom pyraclostrobin is the most commonly used fungicide.

Pyraclostrobin is a new type broad-spectrum methoxy acrylate fungicide that can prevent powdery mildew, rust and downy mildew mainly caused by pathogens of Oomycetes, Ascomycetes and Basidiomycetes [3,4]. Meanwhile, pyraclostrobin can regulate the metabolism of plants, enhance the tolerance of crops in adverse environments and improve crop yields [5,6]. At present, the reports on pyraclostrobin mainly include disease control effects [7], pathogen sensitivity assay [8], toxicity detection and toxicity mechanism of model organisms [9,10], residue detection and dietary risk assessment [11,12]. The residues of pyraclostrobin have been detected in honey, bananas, grapes, citrus and other fruits [13,14,15,16]. Several studies have reported the detection analysis of pyraclostrobin residues with methods such as gas chromatography with mass spectrometry combined with DI-SPME (Direct Immersion–Solid Phase Microextraction), HPLC–MS/MS combined with dispersive solid-phase extraction (DSPE) and modified QuEChERS. Since its appearance, QuEChERS method has been used as a pretreatment for pesticide residues in fruits and vegetables, which has gradually expanded to a larger detection range and matrix, and has become the first choice of the rapid pretreatment technology for pesticide residues due to its rapid and simple characteristics [17,18,19,20]. 

To date, there is little information available about the determination method of pyraclostrobin in *R*. *roxburghii*, as well as the evaluation of its dissipation and residue safety in *R*. *roxburghii* under good agricultural practices (GAP). Aims of this study were (1) to develop a simple and rapid analysis method that can measure pyraclostrobin in *R*. *roxburghii* and soil; (2) to investigate the effects of different spray equipment and formulations on the dissipation behaviors of pyraclostrobin in *R*. *roxburghii*; and (3) to evaluate the degradation dynamics and terminal residues of pyraclostrobin in *R*. *roxburghii* and soil. 

## 2. Materials and Methods

### 2.1. Materials and Reagents

Analytical-grade pyraclostrobin (99.0% purity) and 30% pyraclostrobin SC were provided by Dr. Ehrenstorfer GmbH (Augsburg, Germany) and Jinan Zhongke Bioengineering Co., Ltd. (Jinan, China), respectively. WP containing 20% pyraclostrobin and WG containing 50% pyraclostrobin were supplied by Jiangxi haikuolis Biotechnology Co., Ltd. (Jiangxi, China). Primary secondary amine (PSA) and C_18_ sorbents were purchased from Biocomma Biotechnology Co., Ltd. (Shenzheng, China). Graphite carbon black (GCB) and Florisil sorbents were obtained from Shanghai Aladdin Biochemical Technology Co., Ltd. (Shanghai, China). And LC-grade acetonitrile, methanol, isopropanol and formic acid were obtained from Merck Millipore (Darmstadt, Germany).

A stock standard solution of pyraclostrobin (200 mg/L) was prepared in acetonitrile (LC-grade), and working solutions were prepared by serially diluting the stock solution to obtain concentrations of 5, 2, 1, 0.1, 0.05, 0.01 and 0.001 mg/L. Similarly, *R*. *roxburghii* and soil matrix solution were extracted according to the optimized pretreatment method after the typical blank samples were obtained, followed by the addition of pyraclostrobin standard solution to the blank *R*. *roxburghii* and soil matrix solution to quantitatively prepare matrix matching standard solution of 5, 2, 1, 0.1, 0.05, 0.01 and 0.001 mg/L. All of these solutions were stored at 4 °C until use.

### 2.2. Field Experiment Design

Open-field trials of degradation dynamics of pyraclostrobin were carried out in Longli County (Guizhou Province) during the 2019 and 2020 agricultural seasons (June to September). During the entire experimental period, the average annual temperature of the experimental site was about 16.1 °C and 14.8 °C, the average annual sunshine duration was 1270 h and 1160 h, and the average annual precipitation was 1150 and 1450 mm, respectively. This region belongs to a subtropical monsoon humid climate. The soil type was yellow soil with medium fertility, and the orchard was under conventional management conditions. The *R*. *roxburghii* cultivar “Guinong No.5” was prone to powdery mildew, with an 8-year-old tree and 1.5 m × 2 m row space. The field experiment was designed in accordance with NY/T 788-2018 [21] (Guidelines on Pesticide Residue Trials) issued by the Ministry of Agriculture, P. R. China. The treatments included the degradation of pyraclostrobin in *R*. *roxburghii* fruits by spray equipment and formulations, degradation dynamics and terminal residues and one control plot. There were 6 *R*. *roxburghii* trees in each experimental plot, which was repeated for 3 times. Each plot was separated by the buffer zone to avoid cross pollution. To investigate the effects of different spray equipment and formulations on the dissipation behaviors of pyraclostrobin in *R*. *roxburghii*, there were 3 types of spray equipment used in this study: gaston gasoline piggyback agricultural sprayer (GPAS), manual agricultural backpack sprayer (MABS) and knapsack multi-function electric sprayer (KMES) (30% pyraclostrobin SC was used as the test agent). Spray formulations included 30% pyraclostrobin SC, 20% pyraclostrobin WP and 50% pyraclostrobin WG (only KMES was used as the test spray equipment). For these treatments, pyraclostrobin were sprayed once onto the *R*. *roxburghii* plants with water at 337.5 g of active ingredient per hectare (g a.i./ha), and fruit samples were collected before 2 h of application, as well as after 2 h, 1, 2, 3, 5, 7, 10, 14, 21 and 28 d of application in 2020.The control plot was sprayed with clean water. To evaluate the degradation dynamics of pyraclostrobin in *R*. *roxburghii* and soil, 30% pyraclostrobin SC was sprayed once onto *R*. *roxburghii* and soil at 337.5 g a.i./ha using a KMES in 2019 and 2020. Then, fruit and soil samples were collected with the same interval as the different spray equipment and formulation groups mentioned above. To assess the terminal residues of pyraclostrobin in *R*. *roxburghii* and soil, 30% pyraclostrobin SC was sprayed two or three times every 7 d at a dosage of 225 g a.i./ha (low dosage) and 337.5 g a.i./ha (high dosage) in 2019 and 2020, respectively. Fruit and soil samples were collected after 7, 14, 21 and 28 d of the last application. 

### 2.3. Analytical Procedures

#### 2.3.1. Samples Preparation

*R*. *roxburghii* with the normal growth, no diseases and insect pests, and soil samples (not less than 2.0 kg) were collected randomly from each experimental plot at different intervals. The *R*. *roxburghii* fruit samples were broken with a homogenizer and divided into 500 g subsamples. Soil samples were fully mixed after removing the shrinkage of weeds and stones, and divided into 200 g subsamples. Both *R*. *roxburghii* fruits and soil were stored in a freezer at −20 °C until analysis.

#### 2.3.2. Samples Extraction and Purification

Based on the classical QuEChERS method, the pretreatment method of pyraclostrobin was properly optimized. *R*. *roxburghii* samples were extracted with methanol, acetonitrile, ethyl acetate, dichloromethane, acetonitrile with 1% acetic acid and acetonitrile with 1% ammonia, respectively. The extraction solution was purified with five types of purification agents, including C_18_, PSA, Florisil, C_18_+PSA and GCB. The soil samples were extracted by acetonitrile, acetonitrile with 0.1% formic acid, 1% formic acid, 0.1% acetic acid, 1% acetic acid and 1% ammonia, respectively. The extraction solutions were purified with C_18_, PSA, Florisil and C_18_+PSA, respectively. After the extraction rates of the target compound in *R*. *roxburghii* and soil with different extraction and purification agents were comprehensively compared, the pretreatment method of pyraclostrobin was optimized, then a rapid analysis method of pyraclostrobin in *R*. *roxburghii* and soil was established.

#### 2.3.3. LC–MS/MS Analysis

Pyraclostrobin was separated on a liquid chromatography system (Agilent 1290) tandem mass spectrometry (Agilent G6470A) equipped with positive mode (ESI+) and an Eclipseplus C_18_ column (2.1 × 50 mm, 1.8 µm). The mobile phase consisted of 0.1% formic acid in a mixed solvent of water (A) and acetonitrile (B) with the volume ratio of 70:30. The gradient elution procedure was as follows: 30% B (0–1 min), 70% B (1–3 min) and 30% B (3–5 min). The flow rate of the mobile phase was set at 0.5 mL/min, and the injection volume was 5 µL. The chromatographic column temperature was set at 40 °C and running time was 5 min. The parameters of MS detection were as follows: sheath gas temperature, 250 °C; sheath gas rate, 11 L/min; carrier gas temperature, 300 °C; carrier gas flow rate, 5.1 L/min; capillary voltage, 3500 V; and atomizer pressure, 45.0 psi. The above dry gas, atomization gas, collision gas and sheath gas were of high purity nitrogen (99.99%). The ion pair parameters, fragmentation voltage, collision value and energy parameters of pyraclostrobin are presented in Table 1.

### 2.4. Calculations

#### 2.4.1. Method Validation

According to the SANTE/11813/2017 guidelines [22], the external standard method was adopted for the quantitative analysis before each test. For recovery experiments, different concentrations of spiked samples for pyraclostrobin (0.1, 1 and 5 mg/kg in *R. roxburghii* and soil) were investigated. The precision and accuracy of the analytical method were evaluated by calculating recovery and relative standard deviation (*RSD*) for six replicates. Calibration curves were constructed from six concentration ranges from 0.001 mg/L to 5 mg/L using the correlation coefficient (*R*^2^). The matrix effect (ME) was calculated by the solvent standard curve and matrix matching standard curve as follows:

(1) $$\mathrm{ME}=\frac{K_{m}-K_{S}}{K_{S}}\times 100\%$$

where *K_m_* and *Ks* is the slope of the calibration curves obtained in matrix and pure solvent, respectively. ME = 0 indicates no ME, ME > 0 represents matrix enhancement, whereas ME < 0 denotes matrix inhibition. *LOD* and *LOQ* are defined by signal-to-noise ratios of 3 and 10, respectively [23].

#### 2.4.2. Degradation Kinetics

The first-order kinetic equation was used to evaluate the dissipation of pyraclostrobin in *R. roxburghii* and soil. The specific calculation formula was as follows:

*C*_*t*_ = *C*_0_ × *e^−kt^* (2)

where *C_t_* (mg/kg) denotes the concentration of the compound at time *t* (day), *C*_0_ (mg/kg) represents the initial concentration of the compound.

## 3. Results

### 3.1. Extraction and Purification

**Figure 1:**
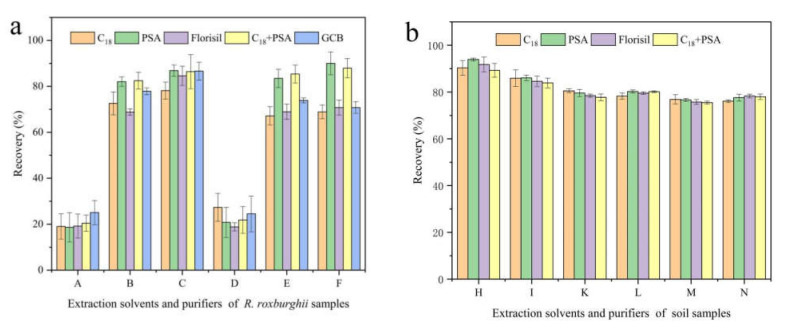
Effects of different extraction solvents and purifiers on the recovery of pyraclostrobin in *R. roxburghii* (**a**) and soil (**b**). The spiked concentration of pyraclostrobin was 1 mg/kg. Note: C_18_, PSA, Florisil, C_18_+PSA and GCB represent purifiers. Letters represent different extraction solvents: A, methanol; B, acetonitrile; C, ethyl acetate; D, dichloromethane; E, acetonitrile with 1% acetic acid; F, acetonitrile with 1% ammonia; H, acetonitrile; I, acetonitrile with 0.1% formic acid; K, acetonitrile with 1% formic acid; L, acetonitrile with 0.1% acetic acid; M, acetonitrile with 1% acetic acid; N, acetonitrile with 1% ammonia.

The commonly used extraction solvents include methanol, acetonitrile, dichloromethane, etc. And the purifiers include PSA, GCB, C_18_ and Florisil [24,25,26,27]. A rapid method for the analysis of pyraclostrobin in *R*. *roxburghii* and soil was established by changing extraction solvents and purifiers and comparing the extraction rates, based on the classical QuEChERS method. As shown in Figure 1, pyraclostrobin in *R*. *roxburghii* was extracted with 1% ammonia acetonitrile solution (*v*/*v*), and the purification of 150 mg AMS plus 50 mg PSA was found to be the best combination. The extraction efficiency of methanol was found to be the worst at less than 10%, followed by dichloromethane, whose the extraction and purification efficiency was less than 30%. For soil samples, acetonitrile solution was used for extraction, and 150 mg AMS plus 50 mg PSA had the best purification efficiency. The recovery reached 93.96%, which was much higher than other extraction solvents and purifiers. Thus, 1% ammonia acetonitrile solution (*v*/*v*) and pure acetonitrile were deemed the best extraction solvents, and PSA was deemed the best purifiers for the recovery of pyraclostrobin in *R*. *roxburghii* and soil samples.

### 3.2. Method Validation

#### 3.2.1. Precision and Accuracy

In this study, pyraclostrobin at concentrations of 0.1, 1 and 5 mg/kg was spiked into the blank *R*. *roxburghii* fruit and soil samples with six replicates to determine the accuracy and precision by intraday variability, which was evaluated by relative standard deviations (*RSD*). As exhibited in Table 2, the recoveries (n = 6) of pyraclostrobin in *R*. *roxburghii* ranged from 90.63% to 105.47% with *RSD* of 1.56~3.18%. The recovery (n = 6) of pyraclostrobin in soil was 94.21~102.38% with corresponding *RSD* between 0.64% and 3.21%. The satisfactory recovery and repeatability demonstrate that this method had superior accuracy and precision, thus it was appropriate for the analysis of pyraclostrobin in *R*. *roxburghii* and soil.

#### 3.2.2. Linearity, Matrix Effect and Detection Limit

The linearity for pyraclostrobin was determined in the concentration ranging from 0.001 to 5 mg/L, and the calibration curves, both in the solvent standard solutions and in the matrix standard solution of the *R*. *roxburghii* and soil, were linear with coefficients of determination (*R*^2^) > 0.99, which indicates a good linear relationship. The obvious matrix effect in *R. roxburghii* and soil was −1.24% and −1.17% respectively, which shows a matrix suppression effect. Hence, the matrix-matched calibration of *R*. *roxburghii* and soil was considered to eliminate the matrix effect in this study. The results indicate that *LOD* of pyraclostrobin in *R*. *roxburghii* and soil was 0.16 and 0.15 µg/kg, and *LOQ* was 0.24 and 0.21 µg/kg (Table 3). Therefore, the sensitivity of the best optimization method met the requirements for detecting pyraclostrobin in *R*. *roxburghii* and soil.

### 3.3. Effects of Spray Equipment and Formulations

**Figure 2:**
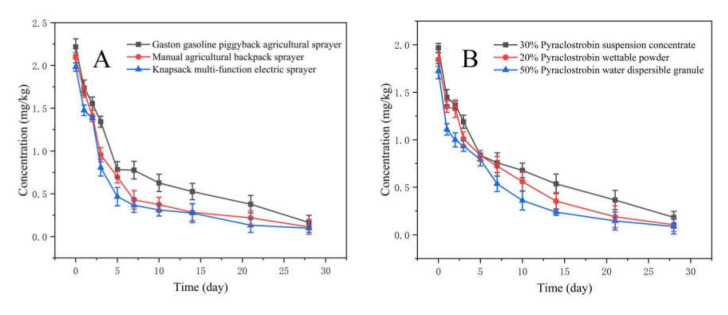
Effects of spray equipment (**A**) and formulations (**B**) on the dissipation of pyraclostrobin in *R. roxburghii*.

The developed method in this study was applied to field-incurred *R*. *roxburghii* samples that had been treated with 30% pyraclostrobin SC. As shown in Figure 2, the initial levels of pyraclostrobin in *R*. *roxburghii* for three types of spray equipment and formulations were as follows: GPAS (2.217 mg/kg) > MABS (2.091 mg/kg) > KMES (1.981 mg/kg) and SC (1.967 mg/kg) > WP (1.843 mg/kg) > WG (1.722 mg/kg). In these cases, levels of residual pyraclostrobin declined rapidly with time. The residues of pyraclostrobin in *R*. *roxburghii* after 28 d of spraying were GPAS (0.164 mg/kg) > MABS (0.113 mg/kg) > KMES (0.098 mg/kg) and SC (0.182 mg/kg) > WP (0.103 mg/kg) > WG (0.085 mg/kg). All residue levels were lower than 0.5 mg/kg (the maximum residue limit (MRL) in kernel fruits recommended by China). The dissipation rate reached 92.60% (GPAS), 94.59% (MABS), 95.05% (KMES), 90.75% (SC), 94.41% (WP) and 95.06% (WG). The data in Table 4 and Table 5 show that the dissipation behavior of pyraclostrobin in *R*. *roxburghii* from different spay equipment and formulations followed the first-order kinetics, and *R*^2^ was greater than 0.93, which indicates that there was a good linear relationship between the test data and the reality results. The half-lives of 30% pyraclostrobin SC in *R*. *roxburghii* were as follows: GPAS (5.38 d) > MABS (3.37 d) > KMES (2.91 d), and SC (6.78 d) > WP (5.64 d) > WG (4.69 d).

The original residues and half-lives of pyraclostrobin in *R*. *roxburghii* were shown as GPAS > MABS > KMES after application with different spray equipment. Owing to the smaller droplet size of KMES, pyraclostrobin could be distributed on the fruits and leaves of *R*. *roxburghii*, which not only had a good control effect against powdery mildew of *R*. *roxburghii*, but also reduced its original residues and half-lives. Compared with other spray equipment under the same conditions, KMES was safer for *R*. *roxburghii*. Meanwhile, the dissipation behavior and degradation rate of the target pesticide were relied on by formulation type. At present, there are few reports about the effect of spray equipment and formulation types on residues of pesticides. The research about formulation types mainly focuses on new pesticide formulation development, such as rapid disintegrating agents [28], tablets, oral films and liquid formulations, in the medical industry [29]. In sum, the spray equipment and formulation types used in this study had an effect on the dissipation of pyraclostrobin in *R*. *roxburghii* and soil under a natural ecological environment. The choice of suitable spray equipment and formulations of pesticides can not only effectively control pests, but also reduce the risk of pesticide residues in agricultural products and the environment. The results here provide appropriate and safe guidance for the application of pyraclostrobin in *R. roxburghii* production.

### 3.4. Degradation of Pyraclostrobin in R. roxburghii and Soil

**Figure 3:**
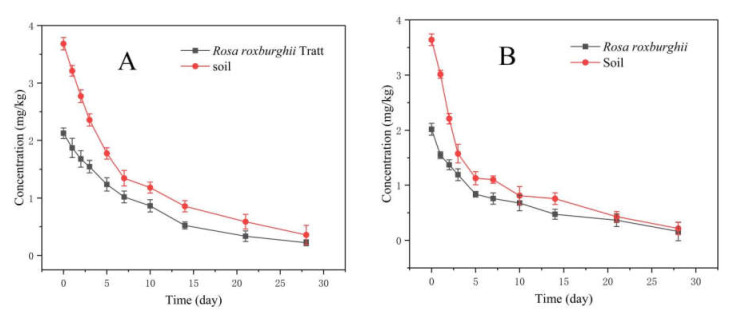
Degradation of 30% pyraclostrobin SC in *R. roxburghii* and soil in 2019 (**A**) and 2020 (**B**).

In 2019 and 2020, the original residues of 30% pyraclostrobin SC in *R*. *roxburghii* were 2.125 and 2.017 mg/kg after 2 h of spraying, respectively. Meanwhile, the original residues of 30% pyraclostrobin SC were 3.684 and 3.640 mg/kg in soil, respectively. The residue amounts at 28 days after spraying were 0.201 and 0.162 mg/kg in *R*. *roxburghii*, 0.357 and 0.215 mg/kg in soil, respectively (Figure 3). As shown in Table 6, the degradation pattern of 30% pyraclostrobin SC in *R*. *roxburghii* and soil followed the first-order kinetics. The half-lives of 30% pyraclostrobin SC in *R*. *roxburghii* were 7.79 and 6.20 d, and 5.95 and 3.86 d in soil, respectively. The decrease of the half-lives in 2020 may result from more rainfall in 2020. In 2019 and 2020, the degradation rate of pyraclostrobin at 28 days after spray was >90% in *R*. *roxburghii* and soil. The reported half-lives of pyraclostrobin were 5.5~8.0 d in blueberries [30], 8.3~9.1 d in bananas [13] and 7.9~15.1 d in apples [31]. The half-lives of the same pesticide in different crops is different, which may vary with different substrates, resulting in different original residues and degradation rates. Meanwhile, some environmental factors, such as temperature, light intensity, rainfall and moisture level, can also significantly affect the dissipation behavior of pesticides [32,33]. In particular, microbes might play an important role in the field soil degradation of pesticides.

### 3.5. Terminal Residues of Pyraclostrobin in R. roxburghii and Soil

As indicated in Table 7, the residue of pyraclostrobin in *R*. *roxburghii* was 0.186~1.236 mg/kg in 2019 and 0.169~1.065 mg/kg in 2020. The residue in *R*. *roxburghii* was less than 0.5 mg/kg at 28 d after the last application. Under the same application conditions, the residue of pyraclostrobin in soil was 0.185~2.996 mg/kg in 2019 and 0.105~3.153 mg/kg in 2020. Pyraclostrobin residues were not detected in *R*. *roxburghii* and soil samples in the control area. The final concentration of pyraclostrobin in *R*. *roxburghii* was <0.5 mg/kg. *R*. *roxburghii* is a kernel fruit; to date, there is no established MRL for residues of pyraclostrobin in *R*. *roxburghii.* However, MRL of other kernel fruits such as apple (0.5 mg/kg) has been established by the Chinese government [34]. Although no health guidance values, such as MRL, are available for pyraclostrobin in *R*. *roxburghii*, its terminal residues in *R*. *roxburghii* in the present study were lower than the officially recommended values. These results provide the Chinese government with data to determine MRL for residues of pyraclostrobin in *R*. *roxburghii*.

Together, the rapid analysis method for residues of pyraclostrobin in *R. roxburghii* and soil as well as its dissipation behaviors and terminal residues in *R. roxburghii* and soil under the field conditions were investigated in this study. The results led from the above-mentioned investigations provide the Chinese government with data to establish MRL for residues of pyraclostrobin in *R*. *roxburghii*, and provide the appropriate and safe guidelines to use pyraclostrobin in the *R*. *roxburghii* cultivation. However, the dietary exposure risk assessment of pyraclostrobin in *R*. *roxburghii* was not involved in the present study. Therefore, further studies are needed to evaluate its safety, such as the identification and analysis of metabolites or the degradation of pyraclostrobin products in *R*. *roxburghii*, as well as to study its metabolic or degradation pathways in *R*. *roxburghii*.

## 4. Conclusions

In this study, a validated QuEChERS and LC–MS/MS analytical method of pyraclostrobin in *R. roxburghii* and soil was developed. This method had satisfactory parameters of higher linearity, accuracy and precision. Subsequently, the dissipation behaviors of pyraclostrobin in *R*. *roxburghii* by different spray equipment and formulations as well as the degradation dynamics and terminal residues in *R*. *roxburghii* and soil were investigated under field conditions. The results show that pyraclostrobin in *R*. *roxburghii* and soil was extracted with 1% ammonia acetonitrile solution or acetonitrile and purified with PSA. The original residues and the half-lives of pyraclostrobin in *R*. *roxburghii* decreased in the order: GPAS > MABS > KMES, and SC > WP > WG. The half-lives of 30% pyraclostrobin SC in *R*. *roxburghii* and soil were 6.20~7.79 d and 3.86~5.95 d, respectively. The terminal residues in *R*. *roxburghii* and soil were 0.169~1.236 mg/kg and 0.105~3.153 mg/kg, respectively. This study provides data to determine MRL of pyraclostrobin in *R*. *roxburghii* as well as provides appropriate and safe guidance to use pyraclostrobin in the production of *R. roxburghii*.

## Figures and Tables

**Table 1:** Mass spectrometric parameters of pyraclostrobin.

Compound	Ionizationmode	Precursor Ion (*m*/*z*)	Production (*m*/*z*)		Fragmentor (V)	Collision Energy (eV)	
Pyraclostrobin	ESI+	388.11	163.0 *	104	94	24	76

Note: Production (*m*/*z*) with * is quantitation.

**Table 2:** The recovery rate of pyraclostrobin in *R. roxburghii* and soil.

Matrix	Additive Concentration (mg/kg)	Recovery (%)							*RSD* (%)
		1	2	3	4	5	6	Average	
**Soil**	**5**	97.33	99.07	97.64	97.56	98.00	97.59	97.86	0.64
	1	99.01	98.79	98.07	99.66	97.15	98.76	98.57	0.88
	0.1	102.38	94.21	97.17	94.69	94.48	97.33	96.71	3.21
*R.* *roxburghii*	5	101.95	102.68	99.25	100.66	98.59	99.97	100.52	1.56
	1	103.95	101.26	104.77	96.58	102.90	105.47	102.48	3.18
	0.1	93.66	96.39	92.84	96.23	91.13	90.63	93.48	2.62

**Table 3:** The matrix linear relationship and matrix effect of pyraclostrobin in different matrices.

Compound	Matrix	Regression Equation	*R* ^2^	*LOD* (µg/kg)	*LOQ* (µg/kg)	ME (%)
Pyraclostrobin	Solvent	*y* = 418305*x* + 8651	0.9969	-	-	-
	Soil	*y* = 403431*x* + 14744	0.9948	0.15	0.21	−1.17
	*R.* *roxburghii*	*y* = 413108*x* + 10481	0.9971	0.16	0.24	−1.24

Notes: *y*, the peak area value; *x*, concentration value; -, blank test with no matrix effect.

**Table 4:** Degradation kinetic parameters of 30% pyraclostrobin SC in *R. roxburghii* by different spray equipment.

Compound	Spray Equipment	Equations	*R* ^2^	Half-Life (d)
30% Pyraclostrobin SC	GPAS	*C_t_* = 2.03405e^−^^0.12877*t*^	0.9416	5.38
	MABS	*C_t_* = 2.05576e^−^^0.20545*t*^	0.9602	3.37
	KMES	*C_t_* = 1.95042e^−^^0.2383*t*^	0.9717	2.91

Note: GPAS, gaston gasoline piggyback agricultural sprayer; KMES, knapsack multi-function electric sprayer; MABS, manual agricultural backpack sprayer.

**Table 5:** Degradation kinetic parameters of 30% pyraclostrobin SC in *R. roxburghii* by different spray formulations.

Compound	Active Ingredient Content	Spray Formulation	Equation	*R* ^2^	Half-Life (d)
Pyraclostrobin	30%	Suspension concentrate	*C_t_* = 1.71653e^−^^0.1021*t*^	0.9356	6.78
	20%	Wettable powder	*C_t_* = 1.66517e^−^^0.12278*t*^	0.9696	5.64
	50%	Water dispersible granule	*C_t_* = 1.50204e^−^^0.14755*t*^	0.9892	4.69

**Table 6:** Degradation kinetic parameters of 30% pyraclostrobin SC in *R. roxburghii* and soil.

Compound	Time	Matrix	Equation	*R* ^2^	Half-Life (d)
30% Pyraclostrobin SC	2019	*R. roxburghii*	*C_t_* = 2.0419e^−^^0.08896*t*^	0.9866	7.79
		Soil	*C_t_* = 3.4721e^−0.11651*t*^	0.9810	5.95
	2020	*R. roxburghii*	*C_t_* = 1.7914e^−^^0.11177*t*^	0.9449	6.20
		Soil	*C_t_* = 3.4169e^−0.17917*t*^	0.9290	3.86

**Table 7:** Terminal residues of 30% pyraclostrobin SC in *R. roxburghii* and soil.

Active Ingredient Concentration (g a.i./ha)	Spray	Interval (d)	Residue (mg/kg)			
			2019		2020	
			*R. roxburghii*	Soil	*R. roxburghii*	Soil
225	2	7	0.701 ± 0.02	1.794 ± 0.02	0.684 ± 0.03	1.928 ± 0.01
		14	0.443 ± 0.04	1.021 ± 0.01	0.452 ± 0.01	0.902 ± 0.02
		21	0.287 ± 0.01	0.544 ± 0.03	0.364 ± 0.04	0.336 ± 0.00
		28	0.186 ± 0.05	0.185 ± 0.06	0.169 ± 0.03	0.105 ± 0.09
	3	7	1.021 ± 0.00	1.981 ± 0.03	0.911 ± 0.02	2.155 ± 0.01
		14	0.754 ± 0.01	1.245 ± 0.05	0.584 ± 0.01	1.388 ± 0.00
		21	0.406 ± 0.02	0.595 ± 0.01	0.334 ± 0.01	0.441 ± 0.03
		28	0.289 ± 0.03	0.187 ± 0.03	0.201 ± 0.05	0.136 ± 0.05
337.5	2	7	1.176 ± 0.03	2.489 ± 0.00	0.836 ± 0.01	2.547 ± 0.00
		14	0.954 ± 0.03	1.521 ± 0.01	0.708 ± 0.00	1.494 ± 0.01
		21	0.667 ± 0.01	0.776 ± 0.03	0.601 ± 0.00	0.467 ± 0.02
		28	0.291 ± 0.05	0.235 ± 0.03	0.266 ± 0.05	0.159 ± 0.07
	3	7	1.236 ± 0.04	2.996 ± 0.03	1.065 ± 0.02	3.153 ± 0.00
		14	1.015 ± 0.01	2.031 ± 0.02	1.002 ± 0.01	1.876 ± 0.00
		21	0.699 ± 0.02	0.965 ± 0.06	0.798 ± 0.01	0.679 ± 0.01
		28	0.481 ± 0.05	0.442 ± 0.02	0.468 ± 0.02	0.383 ± 0.02

## Data Availability

The data that support the findings of this study are available within the manuscript.
